# The intratumoral microbiota biomarkers for predicting survival and efficacy of immunotherapy in patients with ovarian serous cystadenocarcinoma

**DOI:** 10.1186/s13048-024-01464-7

**Published:** 2024-07-05

**Authors:** Hao Qin, Jie Liu, Yi Qu, Yang-Yang Li, Ya-Lan Xu, Yi-Fang Yan

**Affiliations:** 1 State Key Laboratory of Molecular Oncology, National Cancer Center, National Clinical Research Center for Cancer/Cancer Hospital, Chinese Academy of Medical Sciences and Peking Union Medical College, No.17 Panjiayuan Nanli, Chaoyang District, Beijing, 100021 China; 2 Department of Medical Records, Air Force Medical Center, PLA, Air Force Medical University, Beijing, China; 3 State Key Laboratory of Female Fertility Promotion, Center for Reproductive Medicine, Department of Obstetrics and Gynecology, Peking University Third Hospital, No.49 North Huayuan Road, Haidian District, Beijing, 100191 China; 4 National Clinical Research Center for Obstetrics and Gynecology, (Peking University Third Hospital), Beijing, China; 5 Key Laboratory of Assisted Reproduction (Peking University), Ministry of Education, Beijing, China; 6 Beijing Key Laboratory of Reproductive Endocrinology and Assisted Reproductive Technology, Beijing, China; 7 Medical Center for Human Reproduction, Beijing Chao-Yang Hospital, Capital Medical University, Beijing, China; 8 Department of Obstetrics and Gynecology, Peking Union Medical College Hospital, Beijing, China

**Keywords:** Intratumoral microbiota, Ovarian serous cystadenocarcinoma, TCGA-OV, Microbial biomarker, Nomogram

## Abstract

**Background:**

Ovarian serous cystadenocarcinoma, accounting for about 90% of ovarian cancers, is frequently diagnosed at advanced stages, leading to suboptimal treatment outcomes. Given the malignant nature of the disease, effective biomarkers for accurate prediction and personalized treatment remain an urgent clinical need.

**Methods:**

In this study, we analyzed the microbial contents of 453 ovarian serous cystadenocarcinoma and 68 adjacent non-cancerous samples. A univariate Cox regression model was used to identify microorganisms significantly associated with survival and a prognostic risk score model constructed using LASSO Cox regression analysis. Patients were subsequently categorized into high-risk and low-risk groups based on their risk scores.

**Results:**

Survival analysis revealed that patients in the low-risk group had a higher overall survival rate. A nomogram was constructed for easy visualization of the prognostic model. Analysis of immune cell infiltration and immune checkpoint gene expression in both groups showed that both parameters were positively correlated with the risk level, indicating an increased immune response in higher risk groups.

**Conclusion:**

Our findings suggest that microbial profiles in ovarian serous cystadenocarcinoma may serve as viable clinical prognostic indicators. This study provides novel insights into the potential impact of intratumoral microbial communities on disease prognosis and opens avenues for future therapeutic interventions targeting these microorganisms.

**Supplementary Information:**

The online version contains supplementary material available at 10.1186/s13048-024-01464-7.

## Introduction

Ovarian cancer is one of the most common malignant tumors of the female reproductive system worldwide, with 313,959 new cases and 207,252 deaths recorded in 2020 [1]. The past decade has shown a trend of increasing annual incidence. Due to its insidious onset and lack of obvious symptoms in the early stages, 70% patients present with advanced disease at the time of clinical consultation and the reported 5-year survival rate of ovarian cancer is only ~ 30% [2]. The most frequent type of ovarian cancer is ovarian serous cystadenocarcinoma (OSC), accounting for ~ 90% of all ovarian malignancies [3]. Early and adequate screening for OSC should aid in improving patient survival. However, the sensitivity of early disease screening of CA-125, the most commonly used serum biomarker for OSC, in postmenopausal women is only 50–60% [4]. Integration of imaging methods, such as vaginal ultrasound, with combined usage of multiple serum biomarkers, has been shown to improve screening sensitivity for OSC [5]. Therefore, identification of reliable tumor markers is critical for timely diagnosis. In recent years, the relationship between dysregulation of microorganisms and occurrence of different cancer types, including colorectal cancer [6], hepatocellular carcinoma [7], breast cancer [8], and OSC [9], has been a hot topic of research focus. Microorganisms regulate a variety of physiological processes in the host, including immune system activation and metabolic regulation, and thereby play an important contributory role in the occurrence and progression of tumors [10, 11]. The collective findings to date suggest that microbiota within tumors have evolved over time into novel tumorigenesis regulators and could serve as potential biomarkers.

Microbiota regulate carcinogenesis in several ways and influence the tumor microenvironment (TME), including the inflammatory response, thereby affecting treatment outcomes. The importance of the tumor-associated microbiome in cancer immunotherapy is increasingly acknowledged [12]. High-throughput sequencing technology has revealed significantly lower microbial diversity and richness index of OSC relative to normal control tissues [13, 14], suggesting that changes in microbial flora contribute to the incidence and progression of malignancy. In addition, recent reports indicate that changes in gut microflora are closely related to the clinical outcome of OSC, providing guidance for the efficacy of treatments, such as probiotics [15]. Sheng and co-workers [16] reported that tumor microbiota can serve as an independent prognostic predictor of OSC survival. In their study, characteristics of the tumor microbiome had significant prognostic value in OSC patients classified into high- and low-risk groups using the Cox model based on 32 microbes.

In the present study, we investigated the microbiome of OSC and paracancerous tissue samples in the TCGA database, constructed a survival model based on microbial abundance, and identified specific microbial populations associated with prognosis. High abundance of *g_Simiduia*, *g_Halolamina*, *g_Brachymonas*, and *g_Terasakiella* was associated with increased overall survival (OS) times, while *g_Magnetospirillum*, *g_Luteimonas*, *g_Mitsuokella*, *g_Erwinia*, and *g_Salinisphaera* were associated with poor prognosis, supporting the possibility of microbial signatures as prognostic biomarkers in patients with OSC. We further assessed the clinical value of high- and low-risk groups by establishing a nomogram model that integrated clinical factors for prediction of OS probability at 1, 3 and 5 years. The microbiome is considered a secondary genome owing to its significant impact on human health and disease [17]. The current study provides novel insights into the impact of the internal microbiome on disease progression of OSC. The immune system is known to play an important role in cancer and tumor-infiltrating immune cells (TIICs) are one of the main components of the TME. The compositions and functions of TIICs vary with host immune status, indicative of their potential prognostic value [18]. Comparison of immune cell infiltration profiles between high- and low-risk groups of OSC revealed differences in the TME, supporting a critical role of TIICs in tumorigenesis and progression.

In this study, a survival model based on microbial abundance was constructed using TCGA ovarian microbiome data. A Cox regression clinical prediction model was generated based on differences in tumor microbiota and LASSO regression screening variables. A nomogram was drawn and immune cell infiltration and checkpoint gene expression differences related to OSC progression and survival outcomes displayed. Overall, microbiome-based diagnosis of cancer should provide significant clinical benefits along with novel information for further in-depth research into the associations between intratumor microorganisms and malignancies.

## Materials and methods

### Screening and identification of prognosis-associated microbial signatures

The following datasets were obtained from the Cancer Genome Atlas Ovarian Cancer (TCGA-OV) database for analysis: microbiome abundance data (http://ftp.microbio.me/pub/cancer_microbiome_analysis/TCGA/Kraken/Kraken-TCGA-Voom-SNM-Plate-Center-Filtering-Data.csv), microbiome clinical data (http://ftp.microbio.me/pub/cancer_microbiome_analysis/TCGA/Kraken/Metadata-TCGA-Kraken-17625-Samples.csv), expression data (https://gdc-hub.s3.us-east-1.amazonaws.com/download/TCGA-OV.htseq_fpkm.tsv.gz), survival data (https://tcga-xena-hub.s3.us-east-1.amazonaws.com/download/survival%2FOV_survival.txt), and sample phenotype data (https://tcga-xena-hub.s3.us-east-1.amazonaws.com/download/TCGA.OV.sampleMap%2FOV_clinicalMatrix). From the Ensembl database, the human.gtf file (Homo_sapiens.GRCh38.99.gtf.gz) was downloaded (http://www.ensembl.org/info/data/ftp/index.html). The immune cell gene set was obtained from the List of Pan-cancer Immune Metagenes using PubMed Identifier (PMID) 28,052,254. Table 1 presents the clinical features of patients in the TCGA-OV dataset.

### Analysis of OSC tumor and normal tissue microbiota profiles at six taxonomic levels

Microbiome samples were acquired by integrating both genomic and transcriptomic data and pooled according to the TCGA codes for samples. Microbial abundance-related information for each sample was obtained and the average abundance of the populations determined at each taxonomic level: kingdom, phylum, class, order, family, and genus. Boxplots of the top 20 abundant microbial species at each level were generated.

### Identification of specific microbial biomarkers based on linear discriminant analysis effect size (LEfSe) analysis

Using the default parameters, LEfSe analysis was performed on cancer and paracancerous samples to identify differentially expressed microbial species that could potentially serve as biomarkers. LEfSe significance levels were *p* < 0.05 and LDA > 2.0. Since LEfSe analysis needs to input taxa of the species, relevant information for each level of taxonomy is required. Species with no taxonomic information were deleted in advance and only those with complete taxonomic information at each level retained.

## Construction and validation of the microbial prognostic signature

### Univariate Cox regression analysis

Samples of tumors from the TCGA-OV datasets were partitioned into two groups, with 50% allocated for training and the remaining 50% for testing purposes. The R packages survival (v3.2-7) and survminer (v0.4.8) were employed for batch Cox single-factor regression analysis of the abundance values of microbial markers within the training dataset. Microbial markers exhibiting a significant correlation with OS, as indicated by *p* < 0.05, were selected for further evaluation.

### LASSO Cox regression analysis

We conducted additional steps to enhance the dimensionality reduction via LASSO regression of microbial markers associated with prognosis. Subsequently a risk-scoring model was developed for this purpose. The entire approach primarily relied on utilization of the R package glmnet (v4.0-2). To refine the accuracy of the regression model, we initially performed lambda screening via cross-validation. The model associated with the minimum lambda (lambda.min) value was selected, followed by extraction of the abundance matrix corresponding to the microbial markers selected in the model. Subsequently, the risk score for each sample was calculated using the formula: ${\text{R}\text{s}\text{c}\text{o}\text{r}\text{e}}_{i}=\sum _{j=1}^{n} {\text{ abundance }}_{ji}\times {\beta }_{j}$ whereby “abundance” represents the content of the specific microbial marker, “*β*” the regression coefficient (COEF) pertaining to the marker in LASSO regression, and “Rscore” the abundance of significantly relevant markers in each sample, multiplied by COEF of the corresponding marker, and then summed up. Here, “*i*” and “*j*” represent the sample and microbial marker, respectively.

To validate the effectiveness of the model, the median value of the risk scores from the training set was used as the threshold to categorize samples into high- and low-risk groups. This classification system was combined with OS and progression-free interval (PFI) data. Next, Kaplan-Meier survival curves were generated and *p*-values calculated. A *p*-value threshold of < 0.05 was utilized to distinguish between the high- and low-risk groups. Sample risk scores were employed as the prediction results of the model and combined with survival data to compute the area under the curve (AUC). A time-dependent dynamic receiver operating characteristic (ROC) curve was further generated. AUC values for 1-year, 3-year, and 5-year survival were all greater than 0.6, indicating strong performance of the model. To further confirm the robustness of the risk model, we applied it to the verification set, calculating risk scores for samples and generating Kaplan-Meier and ROC curves for this set.

### Nomogram construction

A nomogram was created using the R package rms (v6.1-0) and survival (v3.2-7) to visualize the results of Cox regression. Age, grade, and clinical stage were combined with the function cph to construct a Cox proportional hazards regression model, followed by the function surv to calculate survival probability. Finally, the function nomogram was employed for construction of a nomogram object and plot method for display.

## Microbial population characteristics and host correlations

### Correlation analysis and functional enrichment

To investigate the associations between host gene expression and abundance of characteristic microbial species in the model, an expression matrix of protein-coding genes was constructed. The correlation pairs with |correlation| > 0.2 and *p* < 0.05 were considered significant and subsequently used to generate a correlation heat map. For functional enrichment analysis, we used the clusterProfiler package (v4.6.2) in R, which focused on the filtered protein-coding genes that were significantly associated with microbial markers identified in the model.

### Differential display of immune cell infiltration and checkpoint expression genes

Differential display of immune cell infiltration was achieved using the GSVA R package (v1.34.0) to calculate GSVA enrichment scores for 28 types of immune-infiltrating cells in OSC samples, based on the single-sample gene set enrichment analysis (ssGSEA). Data values were standardized using the scale function and boxplots comparing high- and low-risk groups subsequently generated. For analysis of differences in checkpoint gene expression, a list of 64 target immune checkpoint (ICP) genes was sourced from the existing literature (PMID: 32,814,346) [19]. The differences in expression between high- and low-risk groups were examined and visualized using boxplots to display the distribution of datasets.

## Results

### Study population

A total of 453 samples from the TCGA-OV dataset were utilized (136 patients > 65 years and 317 patients ≤ 65 years). The characteristics of the participants are presented in Table 1.

**Table 1:** Clinical information on TCGA-OV dataset samples. OS, overall survival; GX, Grade could not be assessed

Clinical data of TCGA-OV tumor samples (453)		
Age	> 65	136
	≤ 65	317
Grade	G1-2	58
	G3-4	384
	GX	11
Radiation therapy	YES	3
	NO	380
	NA	70
Clinical stage	Stage I-II	31
	Stage III	354
	Stage IV	65
	unknown	3
OS status	Alive	178
	Dead	263
	unknown	12

## Screening and identification of prognosis-related microbial markers

### Microbiota profiles of human OSC and normal samples

**Fig. 1:**
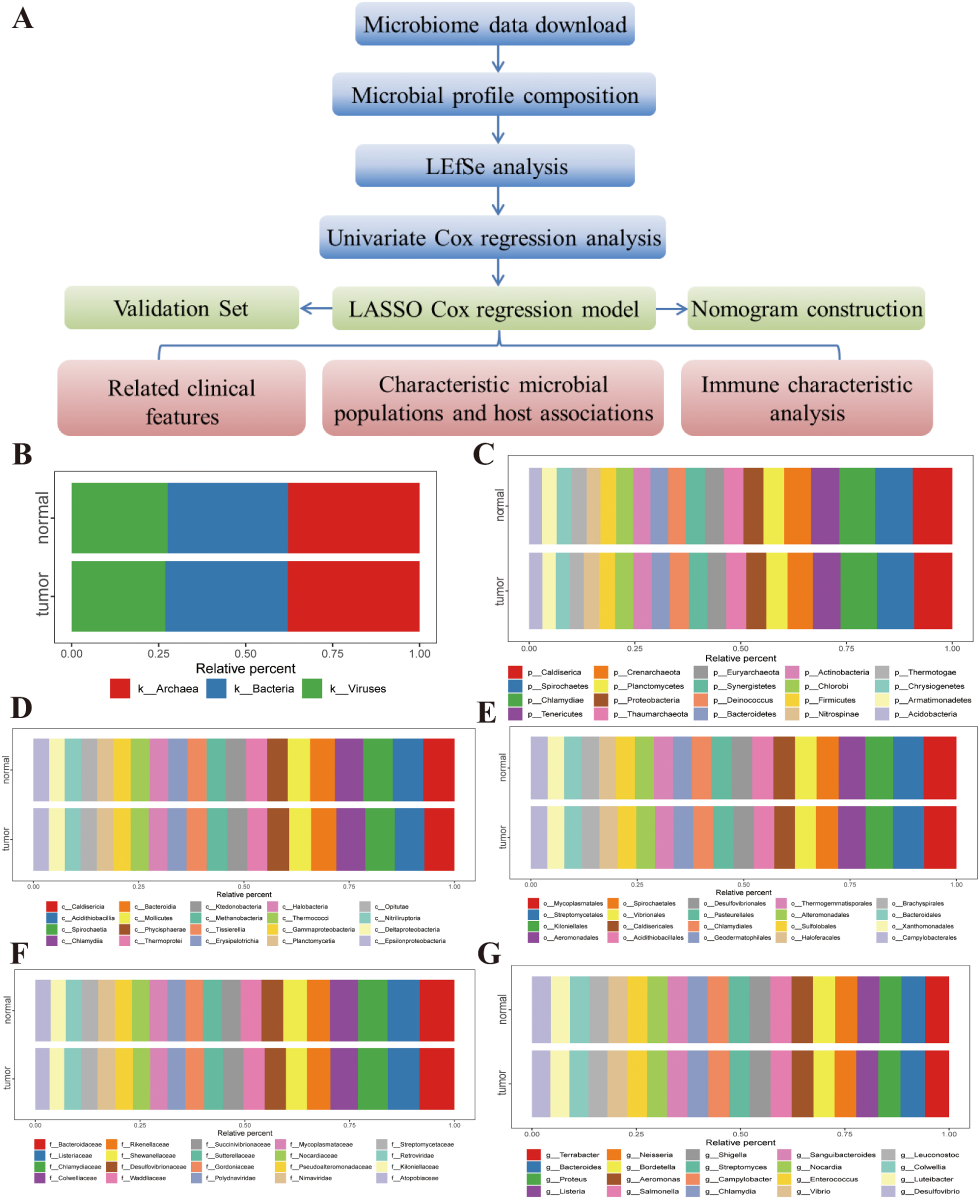
(**A**) Flowchart describing the main design of the current study. (**B**-**G**) Proportions of microbiota in normal and OSC samples distributed according to kingdom, phylum, class, order, family, and genus

Microbial abundance and information on patients with OSC in TGCA (file “Kraken-TCGA-Voom-SNM-Plate-Center-Filtering-Data.csv” and “Metadata-TCGA-Kraken-17625-Samples.csv”) were obtained from the online repository of Poore et al. [20]. Microbiome samples were grouped according to TCGA codes and microbial profiling of relative species composition and abundance at each taxonomic level, specifically, kingdom, phylum, class, order, family, and genus, between normal and OSC tissues summarized. A cladogram was generated using LEfSe to identify major bacteria that could serve as microbiological indicators for distinguishing patients with OSC. To identify tumor prognosis-related characteristics, univariate Cox regression analysis of OS was conducted using R package survival (v3.2-7). Subsequently, a prognostic classifier was generated using a LASSO Cox regression model based on selected microorganisms from these microbial producers. The basic design of this study is depicted graphically as a flowchart (Fig. 1A). Based on the TCGA ID of the sample, microbiomes were merged, and 453 OSC and 68 para-cancer samples finally obtained. Microbiota profiles of tumor and neighboring non-tumor tissues were compared. Next, based on the kingdom, phylum, class, order, family, and genus levels, the average value was taken for merging and the top 20 microorganisms used for display. The results showed that the proportions of each grade were evenly distributed. Moreover, the difference in microbial composition profiles of cancer and paracancerous samples was not significant (Fig. 1B-G).

### LEfSe-based identification of specific microbial markers

**Fig. 2:**
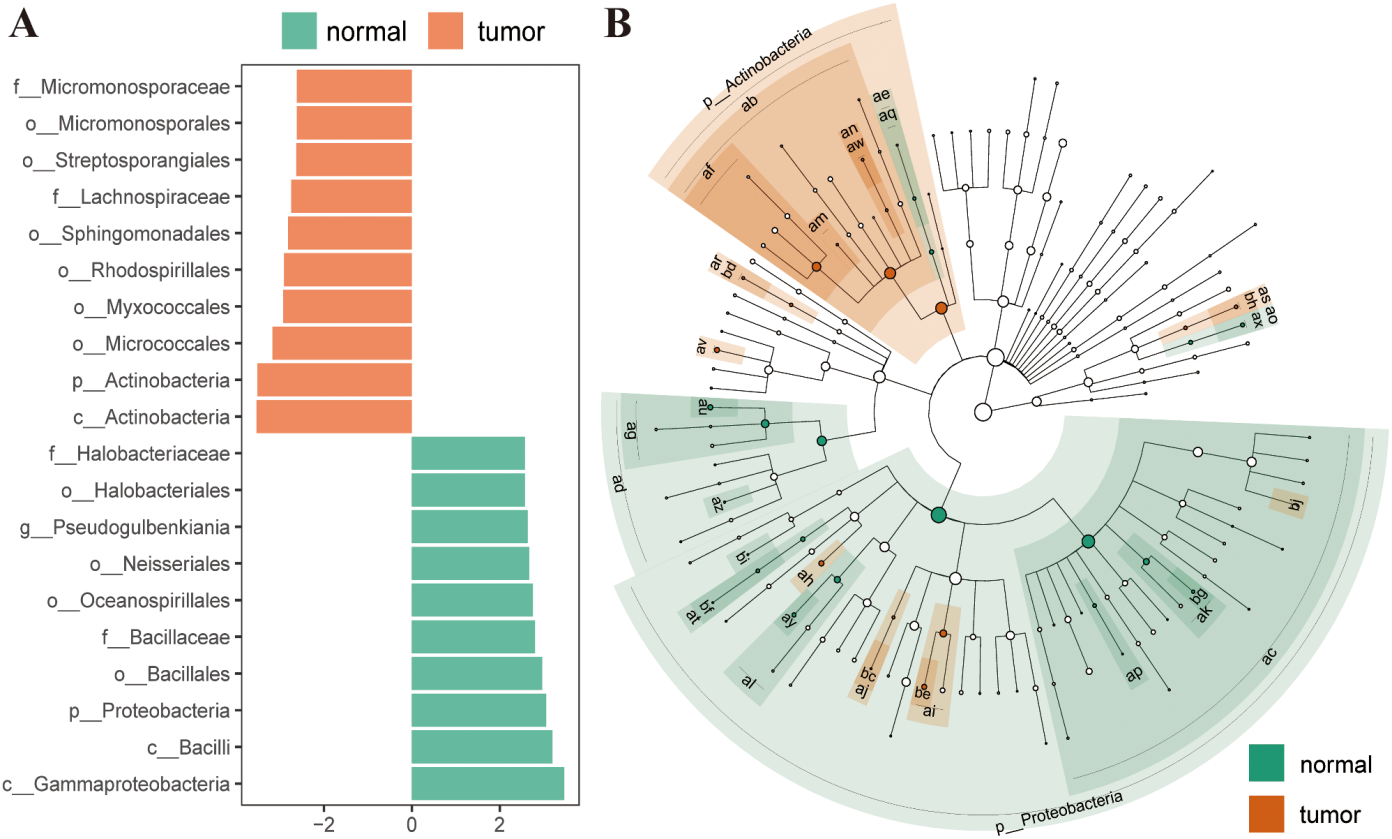
(**A**) Characteristic microorganisms in normal and OSC samples at several taxonomic levels, as determined based on LDA scores from LEfSe analysis. (**B**) Cladogram showing distinct bacterial taxa in OSC and normal groups (grades to genus level)

Based on LEfSe analysis, a total of 308 microbial markers with differential abundance were distinguished between normal and OSC samples, among which 157 had higher linear discriminant analysis (LDA) scores in the tumor group and the remaining 151 had higher LDA values in the normal group. The top 10 relatively abundant microorganisms of each group were selected for display (Fig. 2A, Supplementary Table 1). The evolution clade diagram indicated an important role of *p_Actinobacteria* phylum in the OSC group and *p_Proteobacteria* phylum in the control group (Fig. 2B). Changes in microbial abundance between OSC and normal tissues were detected via computational analysis at the kingdom, phylum, class, order, family, and genus levels, among which 197 differential species were identified at the genus level.

### Construction and validation of a prognostic microbial marker signature

**Fig. 3:**
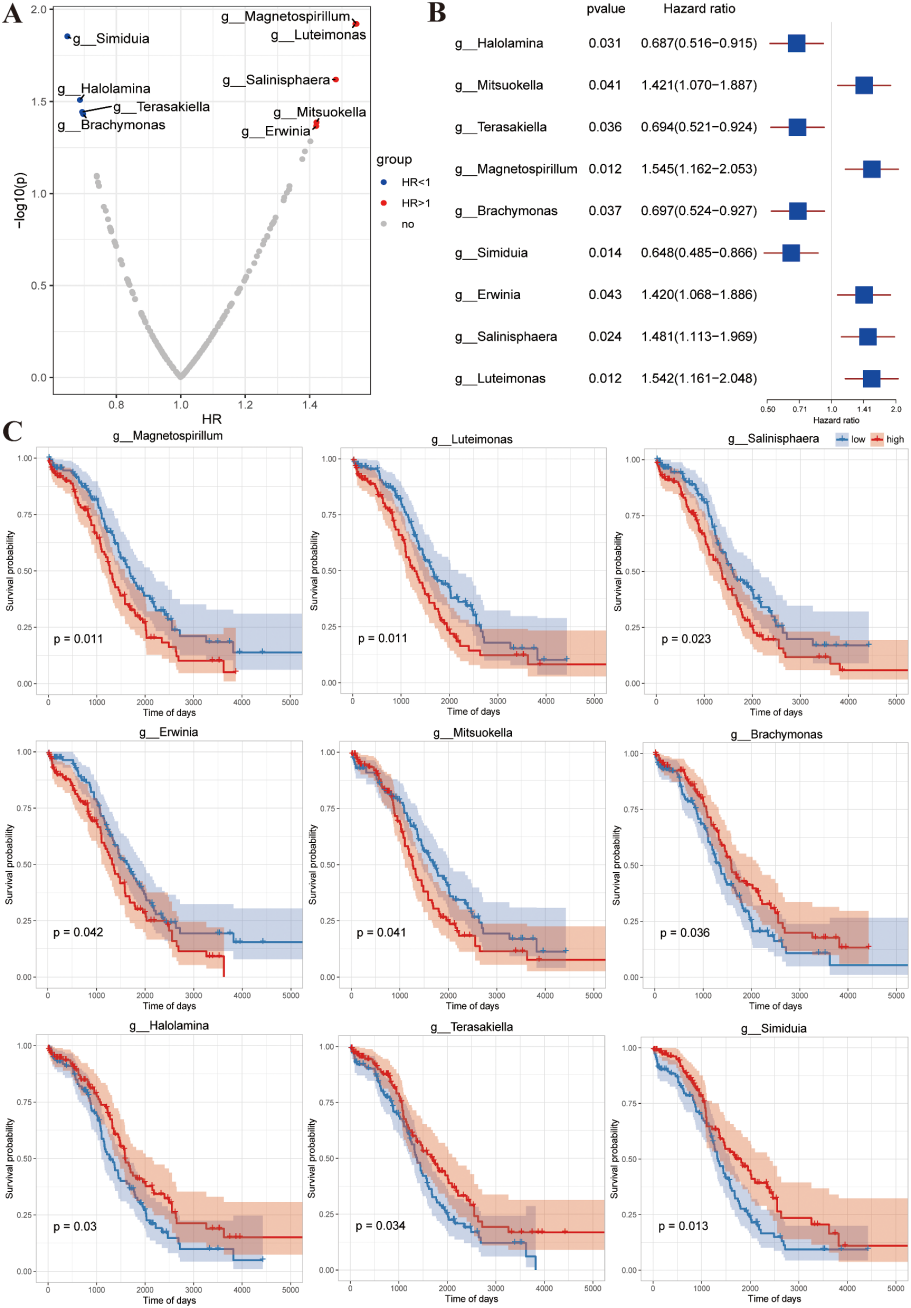
Univariate Cox regression analysis. (**A**) Volcano graph display of abundance-based prognostic value of differential microbial markers. (B) Forest plot depicting univariate Cox regression analysis of microbial risk variables for OS. (C) Kaplan-Meier curves of patients with OSC generated using the top 9 microbial communities (based on *p*-values)

Samples were randomly divided into training and testing datasets at a ratio of 50:50. The total number of samples was 453 (227 in training and 226 in test sets). Based on the abundance of 197 genus-level differential microbial markers combined with OS data, single-factor Cox regression analysis was performed to screen for microorganisms significantly associated with survival. Using the correlation threshold, 9 prognosis-related microbial markers were obtained (*p* < 0.05), of which 5 (*g_Magnetospirillum*, *g_Luteimonas*, *g_Salinisphaera*, *g_Mitsuokella*, and *g_Erwinia*) were identified as risk factors with hazard ratios > 1.0, indicating an association of increased abundance with poor prognosis. The hazard ratios of the remaining 4 microbial markers (*g_Simiduia*, *g_Halolamina*, *g_Terasakiella*, and *g_Brachymonas*) were < 1.0, signifying that reduced microbial abundance was linked to poor prognosis in these cases (Fig. 3A, Supplementary Table 2). Moreover, the above microbial risk factors were included in univariate Cox regression analysis. Our results showed that *g_Mitsuokella* (HR, 1.421; 95% CI, 1.070–1.887; *p* = 0.041), *g_Magnetospirillum* (HR, 1.545; 95% CI, 1.162–2.053; *p* = 0.012), *g_Erwinia* (HR, 1.420; 95% CI, 1.068–1.886; *p* = 0.043), *g_Salinisphaera* (HR, 1.481; 95% CI, 1.113–1.969; *p* = 0.024) and *g_Luteimonas* (HR, 1.542; 95% CI, 1.161–2.048; *p* = 0.012) are prognosis-risk microbes while *g_Halolamina* (HR, 0.687; 95% CI, 0.516–0.915; *p* = 0.031), *g_Terasakiella* (HR, 0.694; 95% CI, 0.521–0.924; *p* = 0.036), *g_Brachymonas* (HR, 0.697; 95% CI, 0.524–0.927; *p* = 0.037) and *g_Simiduia* (HR, 0.648; 95%CI, 0.485–0.866; *p* = 0.014) are prognosis-favorable microbes (Fig. 3B). The top 9 microbes (*g_Magnetospirillum*, *g_Luteimonas*, *g_Salinisphaera*, *g_Mitsuokella*, *g_Erwinia*, *g_Simiduia*, *g_Halolamina*, *g_Terasakiella*, and *g_Brachymonas*) were further applied to generate a Kaplan-Meier curve (Fig. 3C). The prognostic significance of high- and low-abundant microbial markers supports their clinical value in prediction of OSC.

### LASSO Cox regression model of specific microbial markers

**Fig. 4:**
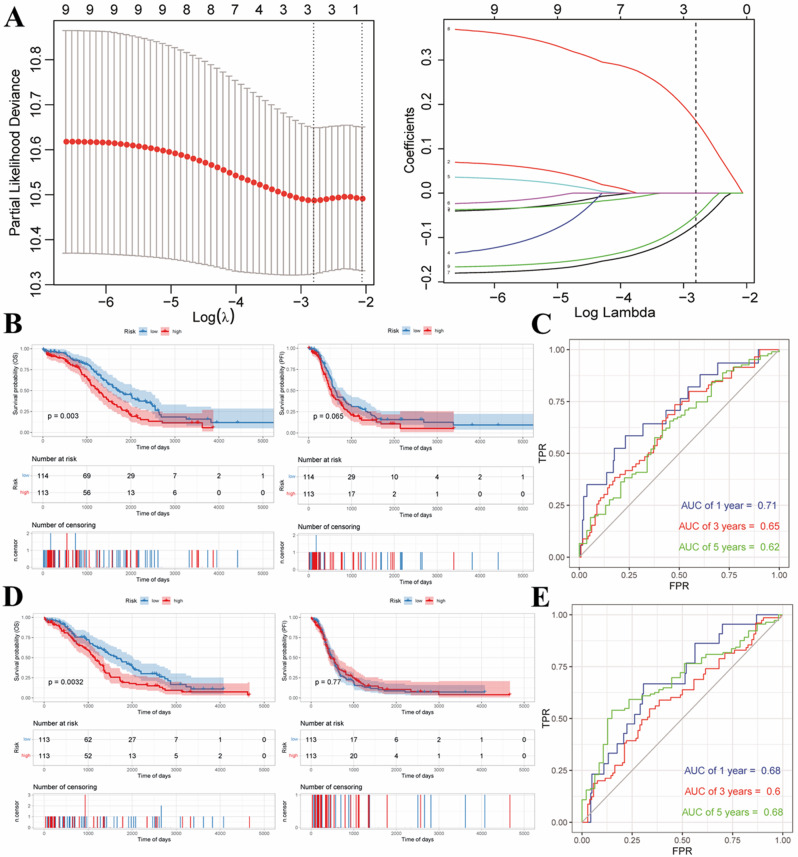
(A) Plots of ten-fold cross-validation error rates and LASSO coefficient profiles of nine microbial markers for OS against the log (lambda) sequence in TCGA-OV. (B, C) Kaplan-Meier and ROC curves of the high- and low-risk categories in the training dataset. (D, E) Kaplan-Meier and ROC curves of the high- and low-risk categories in the validation dataset

We incorporated microbial markers into LASSO Cox regression analysis to develop a prognostic model and calculated the risk scores. LASSO regression was further used to reduce the dimensionality of single-factor Cox regression results. Three microbial markers were obtained for building a risk scoring model (Risk score = *g_Simiduia* * (-0.0711) + *g_Halolamina* * (-0.0511) + *g_Luteimonas* * 0.1634) and sample risk scores calculated (Fig. 4A). Patients were separated into high- and low-risk groups based on median survival times and Kaplan-Meier curves created after integration of the OS data. Survival curves of the two groups were significantly different (*p* < 0.05), but not the Kaplan-Meier curves based on PFI (*p* = 0.065, Fig. 4B). The sample risk score was further used as the prediction result of the model and combined with survival data to calculate area under the curve at 1, 3, and 5 years. All AUC values were greater than 0.6, indicating good predictive ability of the established survival prediction risk score model for OSC patients (Fig. 4C). The performance of the model was confirmed in the validation set and the sample risk scores calculated according to the above formula. Based on the risk scores, groups were divided into high- and low-risk categories using the median value as a threshold and Kaplan-Meier curves subsequently plotted from OS data. The survival curve difference between the two groups was significant (*p* < 0.05), but not the Kaplan-Meier curve based on PFI (*p* = 0.77, Fig. 4D). The sample risk score was utilized as the model prediction result, and the model generated with survival data for 1, 3, and 5 years, with AUC values > 0.6 indicating good performance (Fig. 4E). Our LASSO Cox regression model of the intratumoral microorganism signature performed well on both the training and validation sets.

### Nomogram establishment in conjunction with clinical features

**Fig. 5:**
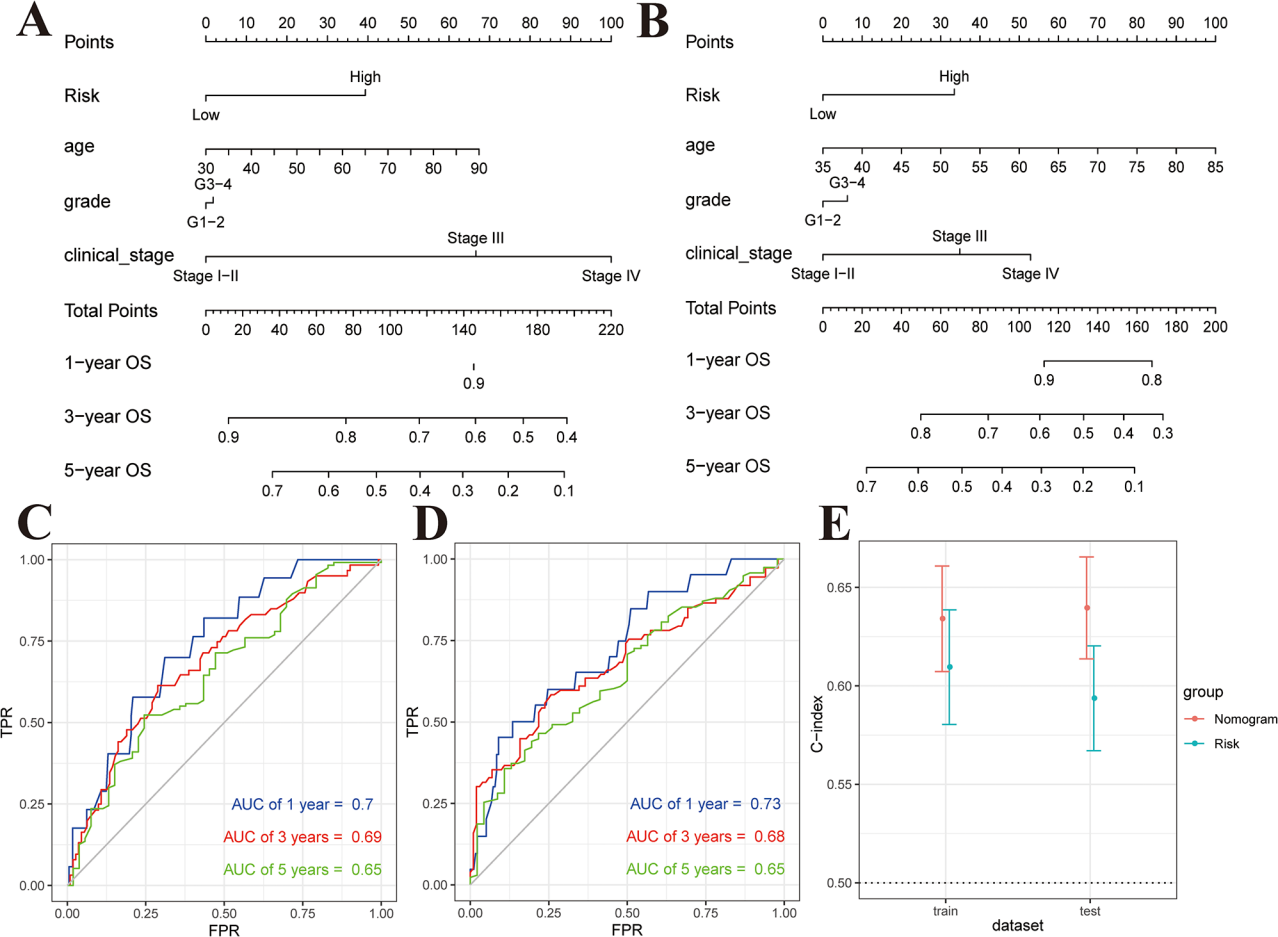
Development and validation of a prognostic nomogram. (A, B) The training and test datasets of patients with OSC were used to construct the prognostic nomogram, allowing for the prediction of 1-year, 3-year, and 5-year OS. (C, D) ROC curves illustrating the performance of the risk model in predicting 1-year, 3-year, and 5-year outcomes in both the training and test datasets. (E) The C-index of the nomogram was assessed in both the training and test datasets, demonstrating its predictive accuracy

The nomogram is a graphical representation of a predictive statistical model for a single patient frequently used as a clinical evaluation tool for prognostic assessment [21]. The nomogram model was built in the training and test datasets using OSC features, such as risk score, age, grade, and clinical stage, and used to predict 1-, 3-, and 5-year OS rates of OSC patients. The results showed that the increased values of clinical risk factors (age, grade, clinical stage, and risk score) were associated with poor prognosis. The risk model offered the most accurate OSC prediction (Fig. 5A, B). Time-dependent ROC curve analysis indicated that the prognostic signature had appropriate accuracy in OS prediction in OSC patients. Moreover, AUC values of the test dataset coincided with those of the training dataset. Regardless of the training or test dataset, the 1-, 3-, and 5-year AUC values were all greater than or equal to 0.65, suggesting good discriminatory value of the nomogram (Fig. 5C, D). In the training and test datasets, the nomogram concordance indexes (C-index) were 0.6340 (95% CI 0.6073–0.6608) and 0.6395 (95% CI 0.6136–0.6654), which were greater than the risk score model C-index values of 0.6095 (95% CI 0.5804–0.6386) and 0.5937 (95% CI 0.5670–0.6203) respectively (Fig. 5E). This finding suggests that the nomogram model of microbial biomarkers taking into account clinical factors is significantly more effective than a single-index model in predicting the prognosis of OSC.

### Characteristic microbial populations and host associations

**Fig. 6:**
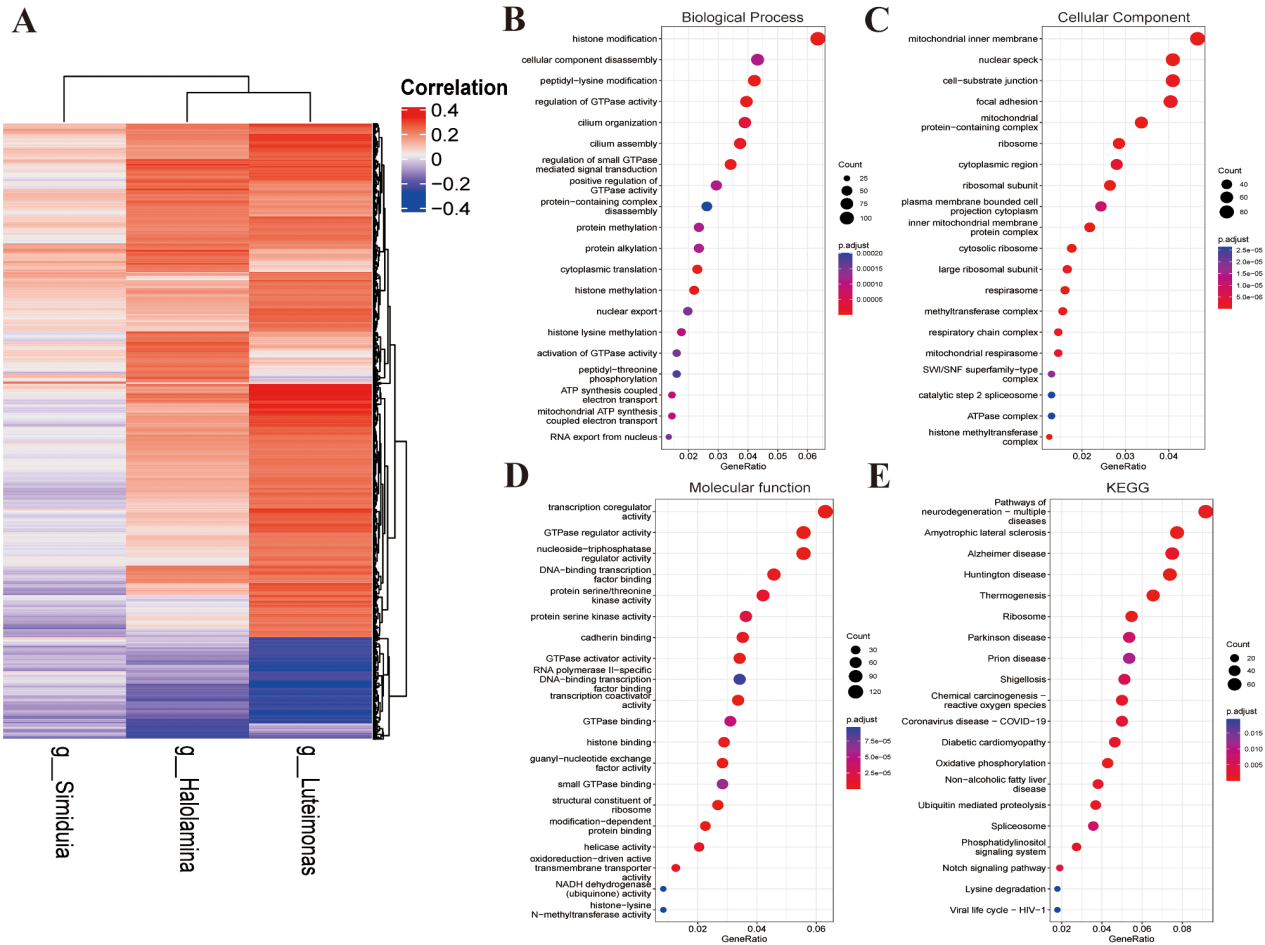
Typical microbial communities and host-microbe relationships. (A) Correlations between host gene expression and microbial species abundance in trait models. (B-E) Functional enrichment analysis of associated genes, including BP, CC, MF, and KEGG pathways

Further statistical analysis of the correlations between abundance of microbial markers in the model and expression of protein-coding genes in the overall sample was conducted. Initially, 9,597 protein-coding genes were identified. Based on the correlation threshold (|correlation| > 0.2 & *p* < 0.05), 2,316 combinations with significant correlations were obtained, including 2,057 protein-coding genes. In the correlation heatmap, *g_Luteimonas* and *g_Halolamina* were significantly positively correlated with the majority of genes and significantly negatively correlated with a small number of genes, while *g_Simiduia* was positively correlated with almost half of the genes and negatively correlated with the other half (Fig. 6A, Supplementary Table 3). Next, host gene functional enrichment analysis was performed on 2057 protein-coding genes. GO enrichment analysis is subdivided into biological process (BP), cellular component (CC), and molecular function (MF) components. BP-enriched pathways were mainly histone modification, cellular component disassembly, and peptidyl-lysine modification, CC-enriched pathways mainly mitochondria inner membrane, nuclear speck, and cell-substrate junction, and MF-enriched pathways mainly transcription coregulator and GTPase regulator activity. KEGG-enriched pathways predominantly included mechanisms of neurodegeneration-multiple diseases, amyotrophic lateral sclerosis, and thermogenesis (Fig. 6B-E).

### Clinical features, therapeutic responses, and immune cell infiltration of the prognostic microbial signature

**Fig. 7:**
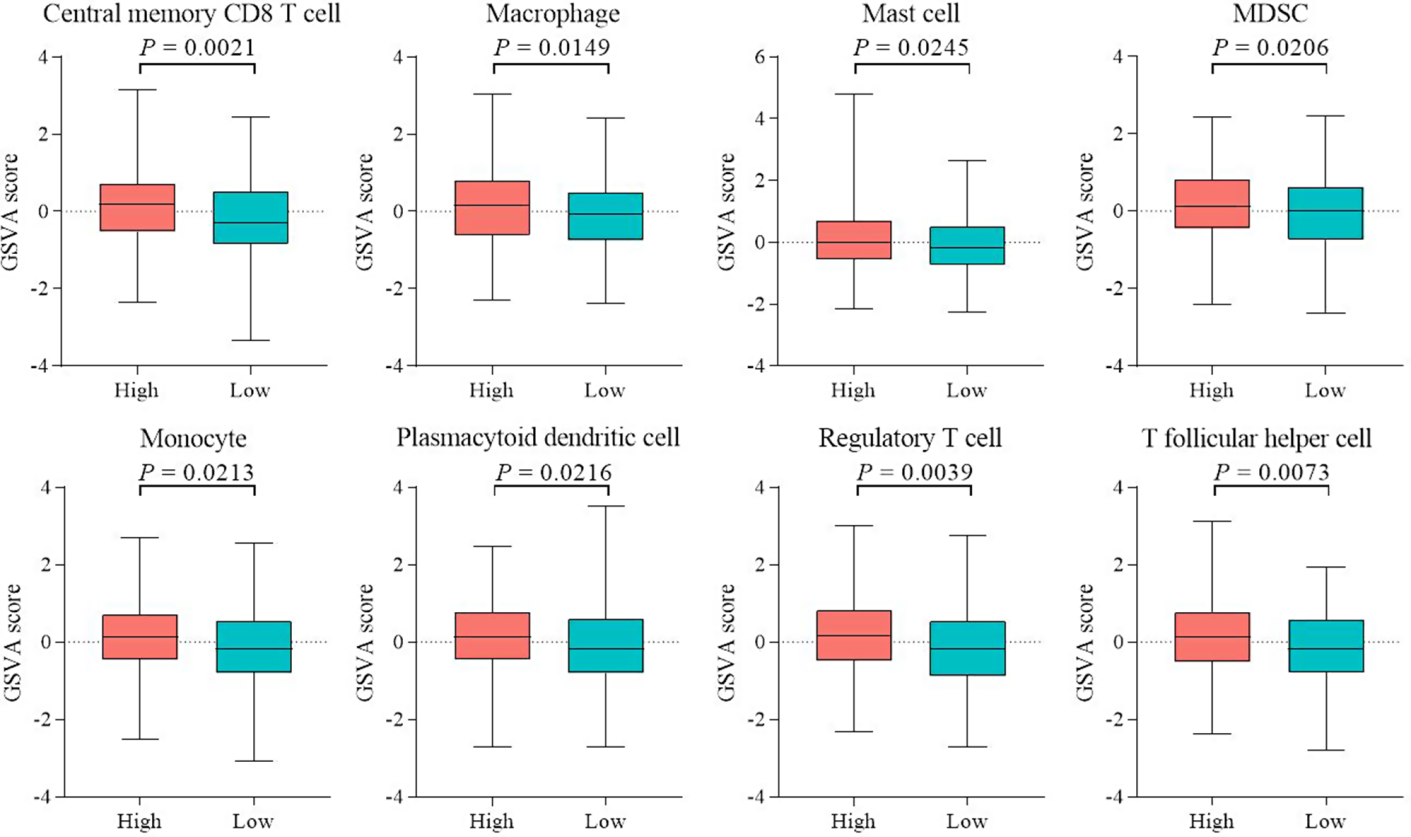
Variations in immunocyte enrichment scores between high-risk and low-risk groups of patients with OSC

Tumor-infiltrating immune cells exert anti-tumor effects through direct killing or secretion of cytokines. These cells are clearly linked to clinical outcomes and therapeutic response in patients with ovarian cancer [22, 23]. In this study, we categorized patients into high-risk and low-risk groups based on the median values of microbial abundance. Subsequently, we conducted GSVA enrichment analysis for both groups and assessed the levels of immune cell infiltration. Notably, our analysis revealed significant disparities in the immunological scores of several immune cell types between the high-risk and low-risk groups, as illustrated in Fig. 7. Significantly higher scores were obtained for central memory CD8 T cells, macrophages, mast cells, myeloid-derived suppressor cells (MDSC), monocytes, plasmacytoid dendritic cells, regulatory T cells, and T follicular helper cells in the high-risk group relative to the low-risk group. This finding is particularly intriguing, given the emerging research showing correlations between immune cell infiltration and OSC. For instance, stromal infiltrating mast cells have been identified as potential indicators of immunoevasive high-grade serous ovarian cancer cases, which are often associated with poorer prognosis and limited responsiveness to immunotherapeutic interventions [24]. Moreover, increased numbers of circulating or tumor-infiltrating MDSCs are reported in ovarian cancer, which are typically linked to poorer prognosis and more advanced clinical stages [25].

### Differential expression of checkpoint immunotherapy marker genes between high-risk and low-risk groups

**Fig. 8:**
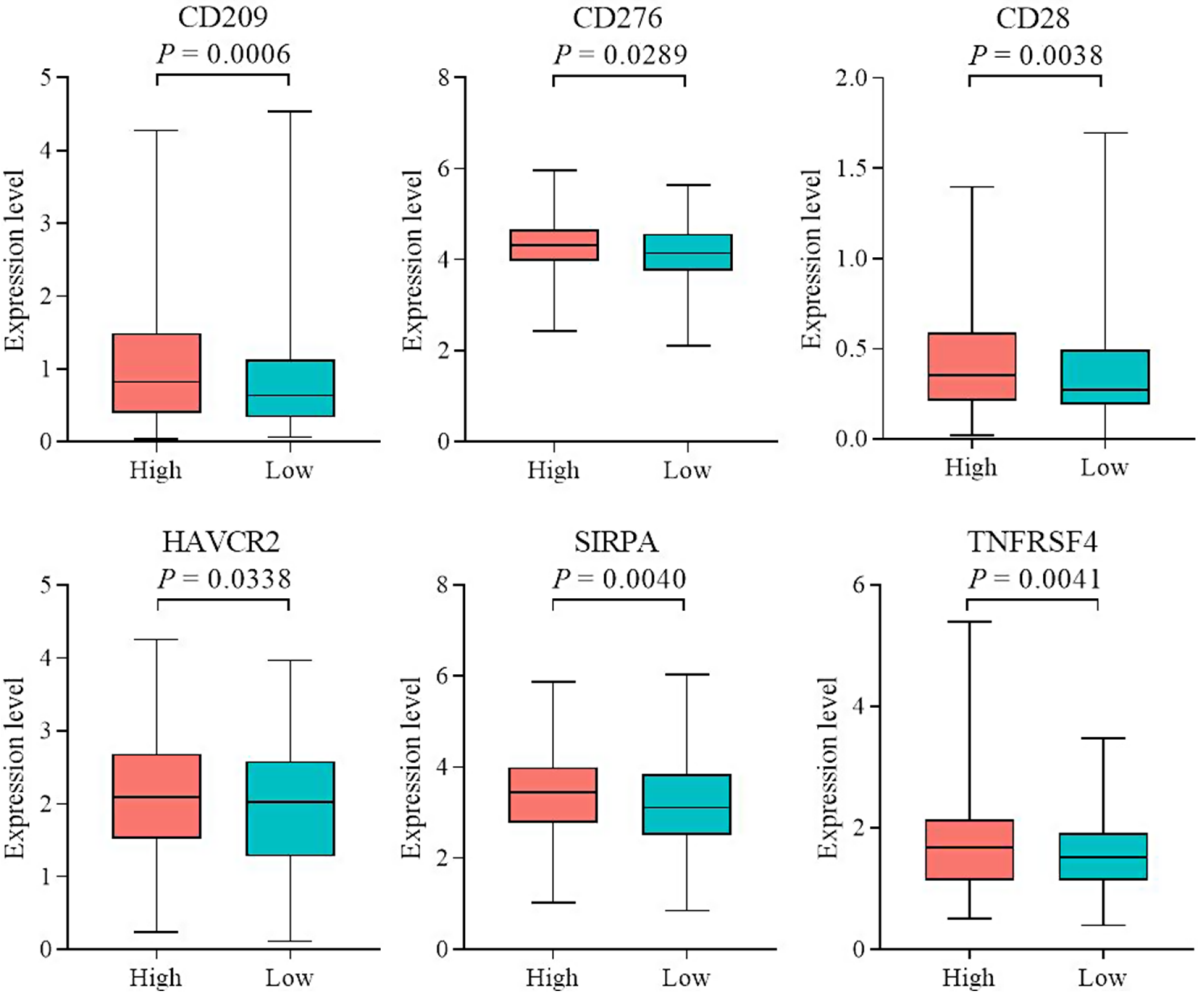
Variations in the expression of immune checkpoint molecules among high- and low-risk cohorts

Expression levels of ICP genes were evaluated in human OSC and control tissues in view of the significant roles of ICP modulators in cancer immunity, which alter the success rates of anticancer treatment [26, 27]. Data on the expression patterns of ICPs in high- and low-risk groups revealed substantial differences in six target genes, including *CD209*, *CD276*, *CD28*, *HAVCR2*, *SIRPA*, and *TNFRSF4*, which displayed significantly higher levels in the high-risk group (Fig. 8). Our findings suggest that in OSC, prognosis and effects of immunotherapy are strongly correlated with ICP expression levels. For example, *CD276* is upregulated in most tumor tissues and belongs to a newly discovered immunoregulatory protein family. Moreover, high expression of *CD276* is associated with poor function of tumor-infiltrating T cells in OC [28, 29]. These substantial variations in ICP molecules between OSC and normal tissues indicate that abnormal ICP expression plays a vital role in the occurrence and development of OSC.

## Discussion

Accumulating evidence supports potentially significant impacts of gut microorganisms on human health, including host immunity, and strong associations of intestinal microbiota imbalance with the incidence of numerous diseases [30, 31]. The intratumoral microbiome is derived from the gut and intestinal microbiota can govern changes in the abundance of microorganisms in tumors. Intestinal microbes infiltrate the tumor site via the circulation, which is a major source of intratumoral microorganisms [32]. Intratumor microbiota can influence tumor growth through multiple processes such as “host-microbiota interactions” and play complex roles [33, 34]. Tumor bacteria are an essential aspect of the TME that are proposed to modify the tumor internal immunological microenvironment via increasing cell mutation rates, modulating signaling pathways, and promoting inflammation, ultimately influencing carcinogenesis and development of malignancy [35–37]. Microbial invasion may induce immune cells to engage in defensive immunological responses. Prolonged inflammation is a risk factor and bacterial communication of metabolites plays a crucial role in pathogenesis of ovarian cancer [9, 38]. Several studies to date suggest that microflora in ovarian cancer are closely related to tumor development. For example, Banerjee et al. [39] demonstrated that the ovarian cancer microbiome is distinct from that of adjacent tissue using the microarray-based approach, PathoChip, for detection of human pathogenic microorganisms. In their study, substantial levels of *Proteobacteria* were detected in non-matched control samples of ovarian cancer, consistent with our findings. Furthermore, several bacterial species highlighted in their research have been associated with the prevalence of other diseases, such as lung and breast cancer [40, 41]. Among the 9 microorganisms related to prognosis in the present study, *Mitsuokella* has been identified as the predominant bacterium of gut microbiota in colorectal cancer patients with metastases [42]. The abundance of *Mitsuokella* genera is significantly correlated with plasma levels of trimethylamine N-oxide (TMAO), which is implicated in promoting carcinogenic processes in colorectal cancer through enhancement of cell proliferation and angiogenesis [43, 44]. Recent advancements in bioinformatics have revealed a role of TMAO in exacerbating ovarian and breast cancer pathogenesis [45]. In this study, we observed an elevated load of *Mitsuokella* in patients with OSC, which was linked to unfavorable prognostic outcomes. One hypothesis to explain this finding is that *Mitsuokella* influences ovarian cancer development through its metabolic production of TMAO, potentially leading to alterations in the intratumoral microenvironment. In the oral microbiota, *Brachymonas* prevalence is markedly lower in patients with colorectal cancer relative to those with colorectal adenomas [46]. Recent research suggests that *Brachymonas* serve as superior biomarkers with utility in identifying the salivary microbiota of healthy volunteers compared to individuals with acute pancreatitis [47]. Our results indicate that increased levels of *Brachymonas* within tumors are associated with improved OS in patients with OSC, consistent with prior research linking higher *Brachymonas* levels to less aggressive disease manifestations. The collective findings to date underscore the potential probiotic role of *Brachymonas*. Further investigation into the prevalence of *Brachymonas* in the oral microbiota of OSC patients could potentially yield novel insights into its therapeutic significance.

The invasion profiles of eight different types of immune cells, including central memory CD8 T cells, macrophages, mast cells, MDSCs, monocytes, plasmacytoid dendritic cells, regulatory T cells, and T follicular helper cells, differed considerably between the high- and low-risk groups in our study. Blockage of immunological checkpoints is a viable technique for increasing anti-tumor immunity in patients, since ICPs are crucial for maintaining self-tolerance and protecting tissues after response to infections [48]. According to a recent study, mRNA and protein levels of hepatitis A virus cellular receptor 2 gene (*HAVCR2*) encoding the potential immune-checkpoint target T-cell immunoglobulin mucin 3 protein are highly upregulated in multiple cancer types, including OSC [49]. The cell surface protein, signal regulatory protein α (SIRPα), features an extracellular region consisting of three immunoglobulin-like domains and an intracellular region [50]. Previous research has shown that the oncolytic virus SG635-SF, engineered to encode the SIRPα-IgG1 Fc gene, effectively reduces the viability of the ovarian cancer cell line SK-OV3. Moreover, SG635-SF was more effective in suppressing the growth of SK-OV3 tumor xenografts through leveraging the combined actions of viral oncolysis and CD47 pathway inhibition [51]. As the predominant inflammatory element within the stroma of numerous tumors, tumor-associated macrophages (TAMs) influence various characteristics of neoplastic tissues. Evidence suggests that TAMs exhibit multiple M2-associated protumoral functions such as suppressing adaptive immunity [52]. It has been demonstrated that the protumoral phenotype of TAMs can be mitigated by suppressing the expression of M2 phenotype markers, CD163 and CD209, in ovarian cancer A2780 cells [53]. Expression levels of potential ICP targets, such as *HAVCR2*, *SIRPA*, and *CD209*, were significantly higher in the high-risk than low-risk patient group in the current study, implying that differences in intratumoral bacteria in ovarian cancer are related to the sensitivity of patients receiving immunotherapy. As an important part of the TME, the intratumor microbiome influences the biological behavior of tumors and specific microbial compositions promote progression of disease. Here, we explored the associations between microbiota and OSC at the microbiological level, providing groundwork for future research on ovarian cancer. The collective findings indicate that the tumor microbiome has significance for clinical prognosis of OSC and provide valuable insights that could aid in the development of strategies for prevention, diagnosis, and treatment of tumors.

Our research addresses novel questions regarding the direct impact of intratumoral microbiota on OSC prognosis and treatment outcomes, specifically in the context of immunotherapy. Through comprehensive analysis of the microbial contents of cancerous and adjacent non-cancerous tissue, we challenge the prevailing hypothesis that cancer dynamics is primarily influenced by gut microbiota. The collective findings suggest that intratumoral microbes play a critical role, which may lead to the development of innovative therapeutic strategies that target these microbial communities to modulate the TME and enhance immunotherapeutic efficacy. This study significantly advances our understanding of role of the microbiome in OSC by establishing that intratumoral microbiota are not merely passive inhabitants but active participants in cancer progression and patient survival. Determination of the correlations of specific microbial profiles with survival rates and immune responses provides valuable insights into the potential mechanisms through which these microbes influence the TME. Overall, this could create new opportunities for the development of microbiome-based diagnostics and therapies that potentially revolutionize the management of ovarian cancer by facilitating more tailored treatment strategies.

The current research presents a unique approach to understanding the process of OSC through integration of intratumoral microbiome profiling with clinical prognosis indicators. Distinct from previous studies, which primarily focused on the broad impact of microbiota on immune responses within the TME [16], we constructed a prognostic risk score model based on microbial abundance in OSC samples from the TCGA database and explored the prognostic significance of specific microbial populations and their direct associations with survival outcomes in OSC. Specific microbial signatures that were significantly correlated with patient survival and immune response modulation were identified. This technique not only refines the predictive accuracy for OSC prognosis but also integrates microbial profiles directly from tumor samples, offering a groundbreaking perspective in the realm of cancer biomarkers. The database utilized in our study primarily permits analysis at the genus level. While this limitation restricts our ability to investigate species-specific relationships, the genus-level data nevertheless provide valuable insights into microbial associations with OSC. The resolution supports the identification of microbial signatures correlated with patient outcomes and immune responses, contributing to our understanding of the role of the tumor microbiome in modulating disease progression and treatment efficacy. Our findings establish the prognostic value of microbial profiles in OSC and their potential significance in disease prognosis and treatment efficacy. Building on these results, isolation of the microbial species identified and in vivo experiments should further validate their functions and interactions with the host immune system. These systematic studies will be crucial for confirming the clinical relevance of specific microbial biomarkers and their therapeutic targeting potential, with the ultimate goal of advancing personalized treatment plans tailored to individual patients.

### Electronic supplementary material

Below is the link to the electronic supplementary material.

Supplementary Material 1

Supplementary Material 2

Supplementary Material 3

## Data Availability

No datasets were generated or analysed during the current study.
